# Povidone Iodine: Properties, Mechanisms of Action, and Role in Infection Control and *Staphylococcus aureus* Decolonization

**DOI:** 10.1128/AAC.00682-20

**Published:** 2020-08-20

**Authors:** Didier Lepelletier, Jean Yves Maillard, Bruno Pozzetto, Anne Simon

**Affiliations:** a Bacteriology/Hospital Hygiene Department, Nantes University Hospital, Nantes, France; b School of Pharmacy and Pharmaceutical Sciences and Cardiff Institute for Tissue Engineering and Repair, Cardiff University, Cardiff, United Kingdom; c GIMAP-EA3064, University of Saint-Etienne, Saint-Etienne, France; d Department of Infectious Agents and Hygiene, Saint-Etienne University Hospital, Saint-Etienne, France; e Pole de Microbiologie, Institut de Recherche Expérimentale et Clinique, Université Catholique de Louvain, Brussels, Belgium

**Keywords:** povidone iodine, nasal decolonization, surgical site infection, *Staphylococcus aureus*

## Abstract

Nasal decolonization is an integral part of the strategies used to control and prevent the spread of methicillin-resistant Staphylococcus aureus (MRSA) infections. The two most commonly used agents for decolonization are intranasal mupirocin 2% ointment and chlorhexidine wash, but the increasing emergence of resistance and treatment failure has underscored the need for alternative therapies. This article discusses povidone iodine (PVP-I) as an alternative decolonization agent and is based on literature reviewed during an expert’s workshop on resistance and MRSA decolonization. Compared to chlorhexidine and mupirocin, respectively, PVP-I 10 and 7.5% solutions demonstrated rapid and superior bactericidal activity against MRSA in *in vitro* and *ex vivo* studies. Notably, PVP-I 10 and 5% solutions were also active against both chlorhexidine-resistant and mupirocin-resistant strains, respectively. Unlike chlorhexidine and mupirocin, available reports have not observed a link between PVP-I and the induction of bacterial resistance or cross-resistance to antiseptics and antibiotics. These preclinical findings also translate into clinical decolonization, where intranasal PVP-I significantly improved the efficacy of chlorhexidine wash and was as effective as mupirocin in reducing surgical site infection in orthopedic surgery. Overall, these qualities of PVP-I make it a useful alternative decolonizing agent for the prevention of S. aureus infections, but additional experimental and clinical data are required to further evaluate the use of PVP-I in this setting.

## Abstract (precis)

Nasal decolonization is an integral part of the strategies used to control and prevent the spread of methicillin-resistant Staphylococcus aureus (MRSA) infections. The two most commonly used agents for decolonization are intranasal mupirocin 2% ointment and chlorhexidine wash, but the increasing emergence of resistance and treatment failure has underscored the need for alternative therapies. This article discusses povidone iodine (PVP-I) as an alternative decolonization agent and is based on literature reviewed during an expert’s workshop on resistance and MRSA decolonization.

## INTRODUCTION

Staphylococcus aureus is a leading cause of health care-associated infections worldwide (1), which include bacteremia, endocarditis, osteomyelitis (2), and surgical site infections (SSIs) (3). S. aureus infections, including those caused by methicillin-susceptible S. aureus (MSSA) and methicillin-resistant S. aureus (MRSA) strains, are associated with prolonged hospital stay and increased mortality (4). S. aureus colonizes several body sites, including the nose, throat, and perineum (5–8). It is estimated that approximately 20% of the general population are permanent nasal carriers of S. aureus (9, 10). Colonization by MRSA increases the risk of infection by up to 27% (11), with infecting strains matching colonizing strains in up to 86% of cases (9, 12).

Despite active surveillance efforts, advances in the prevention of infection and new antibiotics, MRSA remains a prominent pathogen associated with high rates of mortality (2). Decolonization, the goal of which is to decrease or eliminate bacterial load on the body, is an integral part of the strategies used to control and prevent the spread of MRSA (13). This approach involves eradication of MRSA carriage from the nose through the intranasal application of an antimicrobial agent and body washes with an antiseptic soap to eliminate bacteria from other body sites (13). The most commonly used agents for MRSA decolonization are intranasal mupirocin ointment applied to the anterior nares and chlorhexidine body wash (13–15).

Mupirocin nasal ointment is effective in eradicating MRSA colonization in 94% of cases 1 week after treatment and in 65% of cases after longer follow-up (16). Furthermore, mupirocin-based nasal decolonization decreases infections among patients in high-risk settings, including surgery, intensive care unit (ICU), hemodialysis, and long-term care (13, 16). However, there are growing concerns about decolonization failures following the emergence of mupirocin (17, 18) and chlorhexidine resistance (18–20). These concerns, along with suboptimal compliance and the high cost of branded nasal mupirocin ointment, have underscored the need for alternative therapies (15, 21, 22).

We review here the properties, antimicrobial activity, and clinical efficacy of the antiseptic agent povidone iodine (PVP-I) and discuss its role as an alternative agent for S. aureus decolonization.

## METHODS

This narrative review is based primarily on literature reviewed and recommended by the authors based on their expertise and experience in S. aureus decolonization. Additional studies were identified from a search conducted in PubMed using the key terms “povidone iodine” and “Staphylococcus aureus.” Searches in Google Scholar were also carried out using the key terms “povidone iodine,” “Staphylococcus aureus,” and “methicillin-resistant Staphylococcus aureus.” The key terms in all searches were combined using Boolean operators such as “OR” or “AND”. These main searches, carried out in June 2019, aimed to identify full text articles, reporting human studies, published with no date restrictions. Gray literature sources, such as reports, academic dissertations, and conference abstracts, were also examined. The reference lists of included articles were hand-searched to identify any potential relevant articles. A total of 379 English language publications were identified from the PubMed search, and most of these were open-access articles. Papers were selected for inclusion based on their relevance to the topics of this review: the mechanism of action and antimicrobial efficacy of PVP-I, the potential for resistance to PVP-I, and clinical evidence comparing PVP-I to mupirocin and chlorhexidine as a S. aureus decolonization agent, focusing on the efficacy, safety, and cost-effectiveness of these agents in the prevention of SSI.

## POVIDONE IODINE

### Properties.

**FIG 1:**
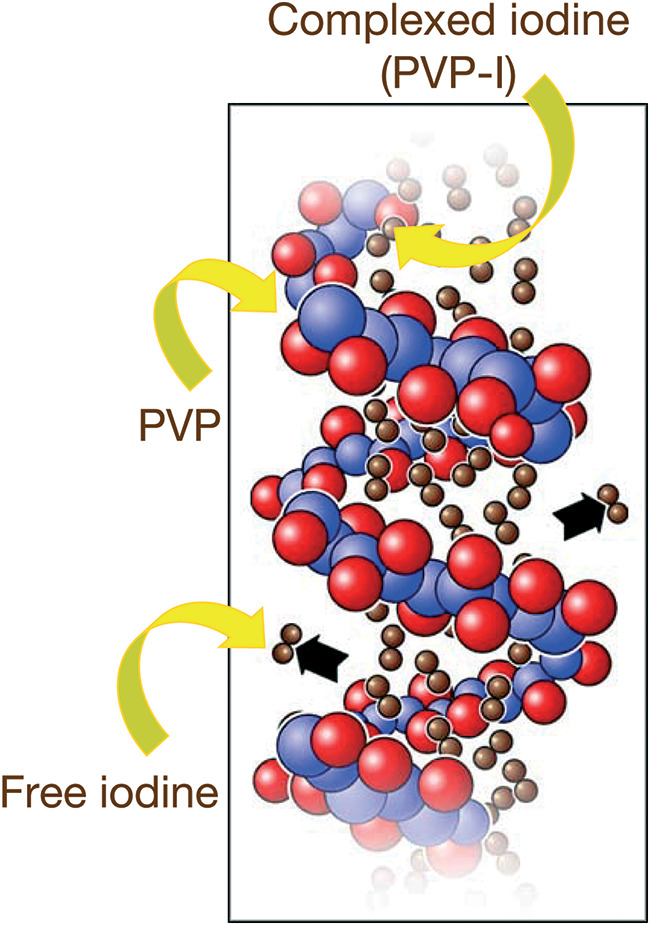
PVP-I (povidone iodine) is a complex of iodine and the solubilizing polymer carrier polyvinylpyrrolidone (PVP) (23, 24). In aqueous solution, a dynamic equilibrium occurs between free iodine and the PVP-I complex (25, 26).

PVP-I is a water-soluble iodophor (or iodine-releasing agent) that consists of a complex between iodine and a solubilizing polymer carrier, polyvinylpyrrolidone (Fig. 1) (23, 24). In aqueous solution, a dynamic equilibrium occurs between free iodine (I_2_), the active bactericidal agent, and the PVP-I-complex. After dilution of PVP-I 10% solution, the iodine levels follow a bell-shaped curve and increase with dilution, reaching a maximum at approximately 0.1% strength solution and then decreasing with further dilution (25, 26). There is a good correlation between free iodine concentration and the microbicidal activity of PVP-I (25, 27).

### Mechanism of action and antimicrobial spectrum.

As a small molecule, iodine rapidly penetrates into microorganisms and oxidizes key proteins, nucleotides, and fatty acids, eventually leading to cell death (23, 24). PVP-I has a broad antimicrobial spectrum with activity against Gram-positive and Gram-negative bacteria, including antibiotic-resistant and antiseptic-resistant strains (28, 29), fungi, and protozoa (Table 1) (23). It is also active against a wide range of enveloped and nonenveloped viruses (30, 31), as well as some bacterial spores with increased exposure time (23). In addition, PVP-I has been shown to have activity against mature bacterial and fungal biofilms *in vitro* and *ex vivo* (32–35).

**TABLE 1:** Indicative antimicrobial spectrum of PVP-I, chlorhexidine, and ethanol^*a*^

Antiseptic	Vegetative bacteria			Spores	Fungi	Viruses
	Gram positive	Gram negative	Actinobacteria			
PVP-I, 10%	BC+++, LS	BC+++, LS	BC++	SC++	FC+++, LS	VC++, LS
Chlorhexidine	BC+++, LS	BC+++, IS	NA	NA	FC++, IS	VC+, IS
Ethanol 70%	BC+, LS	BC+, LS	BC+	NA	FC+, LS	VC+

a Data are as reported by Lachapelle et al. (23), reproduced under CC-BY license. BC, bactericidal; FC, fungicidal; IS, incomplete spectrum (signifying antimicrobial activity is limited to certain, not all, microbes); LS, large spectrum (signifying a broad spectrum of antimicrobial activity); NA, no activity; PVP-I, povidone iodine; SC, sporicidal; VC, virucidal. Strength: +, weak; ++, medium; and +++, high (based on a subjective analysis of eight papers on antiseptic agents by Lachapelle et al.).

### Activity against *S. aureus*.

The activity of PVP-I against S. aureus has been tested using traditional *in vitro* suspension and surface tests, as well as more complex *ex vivo* porcine mucosal and human skin models. It has been suggested that *ex vivo* models may be more clinically relevant, since *in vitro* studies do not take into account host proteins that can neutralize antiseptic activity (36). In contrast, substances commonly present in test media and diluents (e.g., sulfur-containing amino acids) may negate antiseptic activity and lead to false-negative results (37).

The interpretation of *in vitro* studies of PVP-I is also complicated by the paradoxical increase in bactericidal activity with dilutions up to a 0.1% strength solution. This is related to the subtle equilibrium between bound and unbound iodine; the concentration of the latter active compound, I_2_, follows a bell-shaped curve as the dilution is increased. The activity of PVP-I against S. aureus correlates to this, and several studies have observed decreased activity at concentrations above and below 0.1% (25–27, 38–41).

*In vitro* studies have confirmed the bactericidal activity of PVP-I 10% solution against clinical isolates of MSSA and MRSA using both suspension tests (28, 37, 39, 42–45) and surface test methods (46). In these studies, the action of PVP-I against MSSA and MRSA was rapid, with bactericidal activity typically observed within 15 to 60 s (39, 43, 45). Comparative *in vitro* studies have generally showed that, irrespective of exposure time or dilution, 10% PVP-I was more active than chlorhexidine against MRSA and was bactericidal against chlorhexidine-resistant strains (28, 39, 42, 44, 46). Furthermore, two *in vitro* studies also showed that 5% PVP-I cream had bactericidal activity against MSSA, MRSA, and mupirocin-resistant strains of MRSA (40, 43). However, the *in vitro* activity of PVP-I, but not mupirocin, was reduced by the addition of nasal secretions (40).

**FIG 2:**
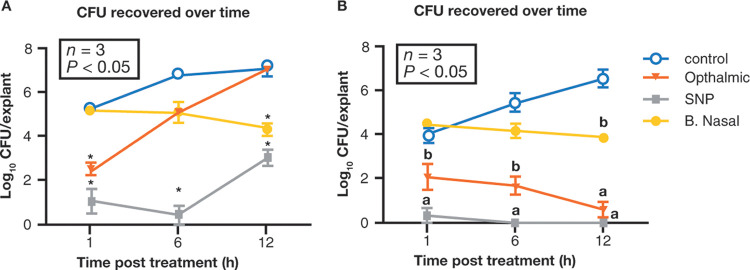
Efficacy of PVP-I (5% ophthalmic solution or 5% SNP), 2% mupirocin (B. Nasal), or no treatment (control) against MRSA infection in *ex vivo* models of porcine vaginal mucosa (A) and human skin (B) (adapted from ref. 47). The results are expressed in log_10_ CFU per explant recovered over time. Values are means ± the standard errors of the means (indicated by error bars). Values that are significantly different (*P* < 0.05) from untreated controls are indicated by an asterisk in panel A. In panel B, values with a different letter (a, b, or no letter) are significantly different (*P* < 0.05) from each other, and values with the same letter are not significantly different (*P* > 0.05) from each other. In panel A, the SNP of 5% PVP-I had significant activity versus the control at all time points, whereas the ophthalmic PVP-I preparation and mupirocin only differed significantly from control at 1 and 12 h, respectively. In panel B, both the SNP and the ophthalmic 5% PVP-I preparations were significantly bactericidal at 1, 6, and 12 h versus the control (*P* < 0.05), while mupirocin only differed significantly from control at 12 h (*P* < 0.05). Labels: B. Nasal, Bactroban nasal ointment; MRSA, methicillin-resistant S. aureus; PVP-I, povidone iodine; SNP, skin and nasal preparation.

In an *ex vivo* model of porcine vaginal mucosa infected with MSSA, 7.5% PVP-I solution significantly reduced bacterial load (measured in log CFU/explant) at 0.25 to 4 h compared to untreated controls, although some regrowth was evident at 24 h (36). In the same porcine model infected with MRSA, a skin and nasal preparation (SNP) of 5% PVP-I significantly reduced viable bacterial cells after 1 h versus the control (1.09 ± 0.57 versus 5.30 ± 0.06 log_10_ CFU/explant, respectively; *P* < 0.05), with sustained activity over 12 h (*P* < 0.05 versus the control) (47). Interestingly, an ophthalmic iodine preparation lacked this sustained activity, only significantly differing from control at 1 h (2.51 ± 0.20; *P* < 0.05). Conversely, mupirocin had a slower onset of action (5.14 ± 0.09 at 1 h, 5.07 ± 0.06 at 6 h; *P* > 0.05), with significant bactericidal effects evident only after 12 h (Fig. 2A) (47). The SNP of 5% PVP-I was also significantly more effective than mupirocin against low-level and high-level mupirocin-resistant MRSA isolates in this porcine model (*P* < 0.05) (47). Finally, in a human skin model infected with MRSA, both the SNP and the ophthalmic PVP-I-based 5% preparation were significantly bactericidal after 1, 6, and 12 h compared to controls (*P* < 0.05), whereas mupirocin was only significantly bactericidal versus control after 12 h (*P* < 0.05) (Fig. 2B) (47).

### Potential for resistance.

As with antibiotics, resistance to antiseptics (the ability of a bacterial strain to survive the use of an antiseptic that could previously eliminate that strain) can occur in bacteria because of intrinsic properties (e.g., biofilm formation, endospores, and expression of intrinsic mechanisms) or can be acquired via mutation or external genetic material (plasmids or transposons) (24, 48, 49). However, less is known about fungal or viral mechanisms of resistance to antiseptics (24, 48). Although there are no standard criteria for evaluating the capability of an antiseptic agent to induce or select for antibiotic resistance in bacteria, a protocol based on evaluating the change in susceptibility profile to the antiseptic and antibiotics has been proposed (50, 51). Based on available reports, no link has been observed between PVP-I and the development of resistance, probably due to its numerous and simultaneous molecular targets (e.g., double bonds, amino groups, and sulfydral groups) (23, 47, 52–56). To date, evidence suggests that PVP-I does not select for resistance among staphylococci (37) and Pseudomonas aeruginosa, Serratia marcescens, Escherichia coli, and Klebsiella aerogenes *in vitro* (28, 57) or among staphylococci after long-term clinical application to catheter exit sites (58). Isolated reports of slow bactericidal activity (27, 41) or apparent resistance to PVP-I (41, 59) are available, but they were later attributed to difficulties in determining its *in vitro* activity and/or the use of culture conditions antagonistic to its action (40). Furthermore, reports of intrinsic contamination of 10% PVP-I solution with *Burkholderia* (formerly *Pseudomonas*) *cepacia* were concerning when initially published in the 1980s and early 1990s (60–66) but were subsequently attributed to confounding factors in the manufacturing process (62). The occurrence of such phenomena has not been widely reproduced with PVP-I, and similar reports of contamination with other antiseptics were also published during this time period (53, 67). Finally, unlike other antiseptics, there have been no reports of PVP-I inducing horizontal gene transfer, antibiotic resistance genes, or cross-tolerance and cross-resistance to antibiotics and other antiseptics (20, 53).

### Clinical evidence.

**(i) Decolonization prior to surgery.** Intranasal and topical PVP-I has been investigated in several studies for the preoperative decolonization of patients undergoing surgery. Four of these studies were prospective with a randomized controlled design (22, 68–70), and the remaining studies were retrospective database studies with historical controls (71–75). The main outcome measures were either S. aureus colonization status (68–70) or the prevention of SSI (22, 71–75). Current World Health Organization (WHO) guidelines for decolonization recommend intranasal mupirocin with or without chlorhexidine body wash in patients with known S. aureus carriage that are undergoing cardiothoracic or orthopedic surgery (1); patients undergoing other types of surgery with known S. aureus carriage should also be considered for decolonization with the same regimen (1). A summary of clinical studies investigating intranasal and topical PVP-I is presented in Table 2 (22, 68–75).

**TABLE 2:** Summary of clinical studies investigating preoperative decolonization with intranasal or topical PVP-I for the prevention of SSIs^*a*^

Treatment and reference	Study design	Patient population	No. of patients	Intervention	S. aureus colonization and SSI rate^*b*^
Intranasal PVP-I					Postoperative nasal colonization
Rezapoor et al. (68)	Randomized, placebo controlled	Orthopedic surgery	34	SNP of 5% PVP-I	7 (21), *P* = 0.003, vs saline
			29	Off-the-shelf 10% PVP-I	15 (52)
			32	Saline (placebo)	19 (59)
					
					SSI rate
Bebko et al. (71)	Retrospective	Orthopedic surgery	365	Intranasal 5% PVP-I (morning of surgery) + 2% CHG wash + 0.12% oral rinse (night before and morning of surgery) versus historical control.	4 (1.1), *P* = 0.02 vs historical control
			344	Historical control (before introduction of decontamination protocol).	13 (3.8)
Urias et al. (75)	Retrospective	Orthopedic surgery	962	Nasal painting with PVP-I skin and nasal antiseptic + CHG washcloth bath/CHG solution shower.	2 (0.2)
			930	CHG washcloth bath/CHG solution shower.	10 (1.1)
Phillips et al. (22)	Prospective, open label, randomized	Orthopedic surgery	842	Two 30-s applications of 5% PVP-I solution into each nostril within 2 h of surgical incision + topical 2% CHG wipes.	6 (NA), *P* = 0.1 vs mupirocin
			855	2% mupirocin ointment twice daily for the 5 days prior to surgery + topical 2% CHG wipes.	14 (NA)
Torres et al. (74)	Retrospective	Orthopedic surgery	1,004	Universal treatment: two 30-s applications of 5% PVP-I solution into each nostril ∼1 h prior to surgical incision + CHG baths for 5 days before surgery + a topical CHG wipe preoperatively applied to the leg.	8 (0.8)
			849	Screening and treatment of MRSA-positive patients: intranasal mupirocin twice daily for 5 days prior to surgical incision + CHG baths for 5 days prior to surgical incision + a topical CHG wipe preoperatively applied to the leg.	6 (0.8)
					
Topical PVP-I					Preoperative skin colonization
Veiga et al. (69)	Prospective, randomized, controlled	Plastic surgery	57	Shower with liquid detergent–based 10% PVP-I 2 h before surgery.	1, *P* = 0.0019 vs control
			57	Control (no special instructions for showering were implemented before surgery).	12
					
					SSI rate
Ghobrial et al. (72)	Prospective database analysis	Spinal neurosurgery	3,185	7.5% PVP-I	33 (1.036), *P* = 0.728 vs CHG/IPA
			3,774	2% CHG and 70% IPA	36 (0.954)
Raja et al. (73)	Retrospective	Cardiac surgery	738	Skin preparation with 10% PVP-I in 30% industrial methylated spirit	NA (3.8), *P* = 0.14
			738	Skin preparation with 2% CHG in 70% IPA	NA (3.3)

a CHG, chlorhexidine; PVP-I, povidone iodine; SNP, skin and nasal preparation; SSI, surgical site infection; IPA, isopropyl alcohol/isopropanol.

b Colonization is expressed as the number of patients with a positive culture result (numbers in parentheses indicate % of patients); the SSI rate is expressed number (%).

**(ii) Efficacy of intranasal PVP-I decolonization.** One large randomized, placebo-controlled study evaluated the effects of intranasal PVP-I on nasal S. aureus colonization in patients undergoing orthopedic surgery (68). In this study, a single preoperative application of 5% PVP-I nasal solution eliminated nasal S. aureus in over two-thirds of patients at 4 h posttreatment, although an off-the-shelf preparation of 10% PVP-I solution was found to be less effective (68). In another randomized, placebo-controlled study, a single application of 10% PVP-I nasal preparation significantly reduced nasal MRSA at 1 and 6 h (*P* < 0.05); however, significant activity was not maintained at 12 and 24 h (*P* > 0.05) (70). Based on these results, the authors concluded that PVP-I may be effective for the short-term suppression of MRSA during surgery, and they proposed that this may be sufficient to reduce the risk of SSI (70). Of interest, the study demonstrated that repeated dosing with PVP-I every 12 h for 5 days did not enhance its efficacy, since there was no significant reduction in nasal MRSA with PVP-I compared to control (*P* > 0.05) (70).

Studies have also investigated the effect of intranasal PVP-I on the rate of SSI. In a retrospective database study, universal preoperative decontamination with intranasal PVP-I plus chlorhexidine wash and oral rinse significantly reduced the 30-day SSI rate versus historical controls in patients undergoing elective orthopedic surgery (1.1% versus 3.8% in controls; *P* = 0.02) (71). In another retrospective review, the addition of intranasal PVP-I to chlorhexidine wash also significantly reduced the SSI rate in trauma patients undergoing emergency orthopedic surgery compared to chlorhexidine alone (0.2% with chlorhexidine plus intranasal PVP-I versus 1.1% with chlorhexidine alone; *P* = 0.02) (Table 2) (75).

Compared to intranasal mupirocin, preoperative intranasal PVP-I was found to have similar efficacy in preventing SSI in patients undergoing orthopedic surgery, with or without screening for MRSA (Table 2) (22, 74). In an investigator-initiated, open-label, randomized study, 90-day deep SSI rates caused by any pathogen, including S. aureus, were similar with either intranasal PVP-I applied within 2 h of surgery or intranasal mupirocin given for 5 days before surgery with topical chlorhexidine (22). Likewise, in a retrospective study, the 90-day SSI rate was similar in patients treated with universal intranasal PVP-I 1 h before surgery compared to screening for MRSA followed by intranasal mupirocin for 5 days (with both groups also treated with chlorhexidine washes and a preoperative chlorhexidine wipe) (74). Alongside similar efficacy, patients treated with mupirocin were more likely to report headache, rhinorrhea, congestion, and sore throat than those treated with PVP-I, in which a single case of vasovagal reaction was reported (22). Overall, fewer patients (3.4%) rated their experience of treatment with nasal PVP-I solution treatment as unpleasant/very unpleasant compared to patients treated with intranasal mupirocin ointment (38.8%) (*P* < 0.0001) (76).

According to a U.S. cost-effectiveness model, universal preoperative decolonization with intranasal PVP-I in patients undergoing orthopedic surgery potentially saved $74.42 per patient compared to preoperative screening and treatment of MRSA-positive patients with a 5-day course of intranasal mupirocin (77). Other studies conducted in different settings led to a similar conclusion, i.e., systemic preoperative decolonization with PVP-I was more cost-effective than the standard MRSA screening protocol in orthopedic surgery, with no difference in infection rates (74, 78).

**(iii) Efficacy of topical PVP-I decolonization.** In a prospective study in patients undergoing elective plastic surgery, a preoperative shower with PVP-I significantly decreased presurgical staphylococcal skin colonization versus controls (*P* < 0.0019) (69). Furthermore, the efficacy of preoperative skin decontamination with topical 7.5 or 10% PVP-I was similar to that of topical 2% chlorhexidine in the prevention of SSI in patients undergoing spinal or cardiac surgery, respectively (Table 2) (72, 73).

**(iv) Decolonization of health care professionals.** In a study investigating the longer-term use of PVP-I in the ICU neonatal setting, intranasal PVP-I cream was applied to health care personnel three times per working day for 3 months. This procedure dramatically reduced the rate of nasal carriage of MRSA from 13.3% at baseline to 0% (79).

## STATE OF THE ART AND PERSPECTIVES

Intranasal mupirocin and topical chlorhexidine are currently the preferred agents for the decolonization of S. aureus. However, the widespread use of mupirocin has led to resistance and treatment failures, and antibiotic resistance and the horizontal transfer of mobile antibiotic resistance elements following low-level exposure to chlorhexidine have also been reported (20, 80), highlighting the need for alternative treatment strategies.

PVP-I has several properties which make it an attractive option for S. aureus decolonization, including strong antistaphylococcal activity in *in vitro* and *ex vivo* models, and uniform activity against S. aureus regardless of the presence of antibiotic or antiseptic resistance (28, 39, 40, 42–44, 46). Of note, a lack of acquired bacterial resistance or cross-resistance to antibiotics or other antiseptics has been observed with PVP-I use (20, 23, 28, 37, 55–58). Furthermore, clinical trial data suggest that intranasal PVP-I demonstrates favorable efficacy in the preoperative decolonization of MRSA and prevention of SSI, compared with chlorhexidine and mupirocin (22, 71, 74). Table 3 summarizes the properties of PVP-I, chlorhexidine, and mupirocin (22, 23, 28, 37, 39, 40, 42–47, 57, 58, 71, 72, 81–101).

**TABLE 3:** State of the art and perspectives: use of PVP-I, CHG, and mupirocin for the preoperative decolonization of MRSA and prevention of SSIs^*a*^

Antiseptic	Spectrum of activity	Activity against MRSA and mupirocin-resistant *S. aureus*	Bacterial resistance	Efficacy in surgical site infections
PVP-I	Broad spectrum, including Gram-positive bacteria, Gram-negative bacteria, actinobacteria, antiviral, antifungal, antiprotozoal, and antispore (23, 81)	• Biocidal against MSSA/MRSA within 15 to 60 s (39, 43, 45)	• Very limited occurrence of the induction of iodine resistance (37, 57, 58)	Preoperative intranasal PVP-I in orthopedic surgery:
		• Active against *S. aureus* regardless of the presence of antibiotic or antiseptic resistance (28, 39, 40, 42–44, 46)	• Does not induce cross-resistance to antibiotics (57, 58)	• Significantly reduced 30-day SSI rate in combination with topical CHG (1.1%) vs controls (3.8%); *P* = 0.02 (71)
				• Similar efficacy (6/842) to a 5-day course of intranasal mupirocin (14/855) in reducing 90-day deep SSI rate following surgery (fraction of surgeries with presence of deep SSI) (22)
Chlorhexidine	Broad spectrum, including activity against Gram-positive bacteria and some Gram-negative bacteria and limited activity against fungi (e.g., yeasts) and enveloped viruses (81)	• Biocidal against MRSA within 2 to 30 min (45)	• Several reports of the induction of CHG resistance but a low incidence in some studies (81, 84–90)	Preoperative topical CHG in orthopedic surgery:
		• Dual CHG and mupirocin-resistant MRSA rare but has been reported to cause decolonization failure (82)	• More common in MRSA than MSSA (91)	• 2% CHG no-rinse cloth reduced SSI rates from 3.19% to 1.59% when introduced into decolonization protocol in orthopedic surgery (93)
		• One study found that in mupirocin-resistant MRSA, the rate of *qacA/B* gene was 65% and *smr* gene was 71% (83)	• Reports of cross-resistance to antibiotics (92)	• No significant difference in incidence of SSI between topical 2% CHG (36 [0.954%] of 3,774) and topical 7.5% PVP-I (33 [1.036%] of 3,185; *P* = 0.728) (72)
Mupirocin	Broad antibacterial spectrum, including some Gram-positive bacteria (staphylococci and streptococci) and some Gram-negative bacteria (*Haemophilus influenza*e, *Neisseria* spp., and *Branhamella catarrhalis*) (94)	Active against MRSA after 12 h (47)	• Several reports of resistance (95–99)	Intranasal mupirocin in orthopedic surgery:
				• No significant difference in SSI rate between nasal mupirocin (3.8%) and placebo (4.7%) (100)
			• High-level resistance (MIC ≥512 μg/ml) associated with treatment failure (95)	Intranasal mupirocin in other surgery (gynecologic, neurologic, or cardiothoracic surgery):
			• Clinical significance of low-level resistance (MIC ≥8–256 μg/ml) unknown (95)	• No significant difference in *S. aureus* SSI rate between nasal mupirocin (2.3%) and placebo (2.4%) (101)

a CHG, chlorhexidine gluconate; MRSA, methicillin-resistant S. aureus; MSSA, methicillin-sensitive S. aureus; PVP-I, povidone iodine; qac, quaternary ammonium compound; *smr*, streptomycin resistance gene; SSI, surgical site infection.

Decolonization is most effective among patient populations who are at risk of infection for only a short period (13). As documented in the WHO guidelines (1), the strongest evidence for decolonization is among surgical patients to prevent postoperative SSIs, particularly those undergoing cardiac and orthopedic surgery (13). Overall, the clinical data published to date on intranasal PVP-I support its short-term use prior to orthopedic surgery. Its successful use in trauma patients undergoing emergency surgery within 24 h of admission is also notable, since the use of a 5-day regimen of mupirocin is not feasible in this setting (75). Recent data indicate that a single preoperative application of intranasal PVP-I may be enough for the short-term suppression of MRSA during the perioperative period (70). Therefore, repeated dosing with intranasal PVP-I after surgery in order to enhance or prolong its antimicrobial activity may not be necessary, although studies are required to investigate this further. A large, randomized, controlled clinical trial is currently recruiting patients in order to compare the efficacy and safety of alcohol-based solutions of 5% PVP-I and 2% chlorhexidine in reducing SSI after cardiac surgery (102). Such a study should help to address the paucity of data regarding preoperative decolonization with topical PVP-I versus chlorhexidine for the prevention of SSI in patients undergoing cardiac surgery. Moving forward, studies investigating PVP-I in other patient groups who are at an elevated risk of infection for short periods (e.g., ICU patients) would be of interest.

The management of MRSA colonization continues to evolve, and decolonization should not be considered in isolation. Other notable preventative measures include screening, contact isolation, environmental disinfection, and good hand hygiene practices. Usually, a collection of interventions works better than just one, and combined interventions can reduce infection rates by 40 to 60% (2). With this in mind, further research is required to define the best approaches for persistent carriers, as well as the efficacy of different decolonization strategies and protocols in both surgical and nonsurgical patients (1, 2, 13).

Despite the wealth of evidence obtained from different *in vitro* and *ex vivo* settings supporting the use of various concentrations of PVP-I (5, 7.5, and 10%), the selection of the most appropriate concentration of PVP-I for clinical use should be made on a case-by-case basis. One factor to consider that may guide selection is whether PVP-I is available in aqueous or alcoholic solution. For example, based on our clinical experience, we advocate 10% PVP-I in aqueous solution for use on mucous membranes, whereas we recommend 5% PVP-I in alcoholic solution for use on healthy skin before an invasive or surgical procedure. The final selection should be made by the treating physician, with the ultimate goal to select the concentration of PVP-I which will significantly reduce the bacterial load of the skin without causing issues with skin toxicity.

## FUTURE AREAS OF RESEARCH WITH POVIDONE IODINE

Studies investigating the longer term effects of PVP-I (e.g., prevention of recolonization postsurgery and use in long-term-care facilities) are needed. We also recognized the paucity of information related to bacterial resistance to PVP-I. For this reason, studies examining the potential for bacterial resistance to PVP-I are also recommended, as well as studies to confirm the absence of an association between exposure to PVP-I and the selection of antibiotic resistance among recent clinical isolates.

Evidence suggests that PVP-I in combination with chlorhexidine may prove to be more effective in preoperative antisepsis than when either agent is used alone, a finding indicative of a possible synergistic effect between the two agents (103–105). Indeed, given the different mechanisms of action of PVP-I and chlorhexidine, there is good reason to believe that the disruptive action of chlorhexidine on the bacterial cell membrane may facilitate intracellular entry of PVP-I, thereby potentiating its antimicrobial efficacy (104). A synergistic effect with the use of two or more antimicrobials would provide the opportunity to reduce the dose of each respective antimicrobial, helping to further minimize possible adverse effects without sacrificing antimicrobial activity. Overall, there is a general absence of data relating to the possible synergistic actions of PVP-I in combination with other antiseptics or antibiotics, and this is an area of research that warrants further investigation.

Some studies suggest that PVP-I may have a shorter time to bacterial regrowth than other antiseptic agents (36, 68, 70, 106, 107). Although this is unlikely to be an issue for surgical patients, clinical studies are needed to understand the long-term dynamics of PVP-I. In the case of S. aureus, the bacterium is capable of invading nasal epithelial cells, which appears to protect it from host defense mechanisms (7). A better understanding of the role of the resulting intracellular reservoir of S. aureus during nasal colonization may lead to improved decolonization procedures. This is necessary since both mupirocin and chlorhexidine exhibit weak activity against intracellular S. aureus (108), and there are currently no data available for PVP-I.

## CONCLUSIONS

Based on current evidence, PVP-I may be a useful preoperative decolonizing agent for the prevention of S. aureus infections, including MRSA and mupirocin-resistant strains. The broad spectrum of activity of PVP-I, encompassing viruses and fungi, and its reported activity against biofilm formation distinguish it from other antiseptics. However, compared to the current literature, additional experimental and clinical data are required to further evaluate the use of PVP-I in this setting.
